# Supplementary material for: Preleukemic single-cell landscapes reveal mutation-specific mechanisms and gene programs predictive of AML patient outcomes
Source: Cell Genom. 2023 Oct 27;3(12):100426. doi: 10.1016/j.xgen.2023.100426 (PMC10726426; doi:10.1016/j.xgen.2023.100426)
Supplement: Document S1. Figures S1–S12 [file mmc1.pdf]

Cell Genomics, Volume *3*

Supplemental information

Preleukemic single-cell landscapes reveal mutation-specific mechanisms and gene programs predictive of AML patient outcomes

Tomoya Isobe, Iwo Kucinski, Melania Barile, Xiaonan Wang, Rebecca Hannah, Hugo P. Bastos, Shirom Chabra, M.S. Vijayabaskar, Katherine H.M. Sturgess, Matthew J. Williams, George Giotopoulos, Ludovica Marando, Juan Li, Justyna Rak, Malgorzata Gozdecka, Daniel Prins, Mairi S. Shepherd, Sam Watcham, Anthony R. Green, David G. Kent, George S. Vassiliou, Brian J.P. Huntly, Nicola K. Wilson, and Berthold Göttgens

Supplemental Information

Preleukemic single-cell landscapes reveal mutation-specific mechanisms and gene programs predictive of AML patient outcomes

Tomoya Isobe[1], Iwo Kucinski[1], Melania Barile[1], Xiaonan Wang[1], Rebecca Hannah[1], Hugo P. Bastos[1], Shirom Chabra[1], M. S. Vijayabaskar[1], Katherine H. M. Sturgess[1], Matthew J. Williams[1], George Giotopoulos[1], Ludovica Marando[1], Juan Li[1], Justyna Rak[1,2], Malgorzata Gozdecka[1,2], Daniel Prins[1], Mairi S. Shepherd[1], Sam Watcham[1], Anthony R. Green[1], David G. Kent[1,3], George S. Vassiliou[1,2], Brian J. P. Huntly[1], Nicola K. Wilson[1,4,*], Berthold Göttgens[1,4,*]

Inventory of Supplemental Information

Figure S1 (related to Figure 1). Reference-based data integration

Figure S2 (related to Figure 2). Differential abundance in preleukemic mutant models

Figure S3 (related to Figure 3). Biased cellular fate probability in preleukemic mutant models

Figure S4 (related to Figure 4). Literature-based validation of transcriptome-based metabolic profiling

Figure S5 (related to Figure 5). Consistent gene expression changes in mouse models and human patients

Figure S6 (related to Figure 5). Defining the lineage trajectories for pseudotemporal gene expression analysis

Figure S7 (related to Figure 1-5). Preleukemic perturbations in *Tet2* KO mouse HSPCs

Figure S8 (related to Figure 6). Clustering of TCGA AML patients

Figure S9 (related to Figure 6). Significant upregulation of 12 PLPS genes in the Stem cluster

Figure S10 (related to Figure 6). PLPS and Stem11-based characterization of AML patient cohorts

Figure S11 (related to Figure 6). Mutational landscapes of Stem11 high and low patients

Figure S12 (related to Figure 1-6). Flow cytometry sorting of mouse HSPCs

Figure S1

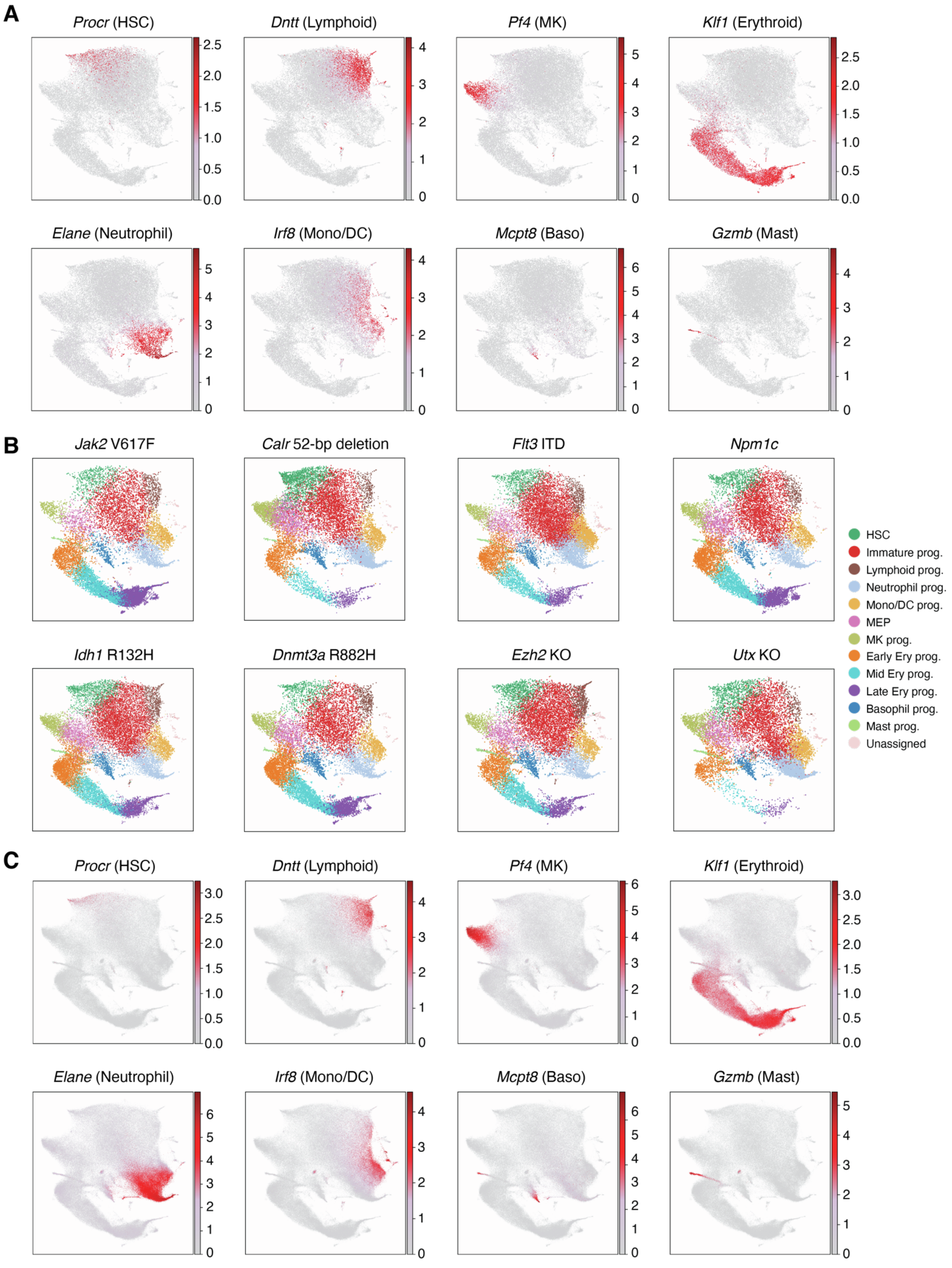
A
Procr (HSC)
Dntt (Lymphoid)
Pf4 (MK)
Klf1 (Erythroid)
Elane (Neutrophil)
Irf8 (Mono/DC)
Mcpt8 (Baso)
Gzmb (Mast)
B
Jak2 V617F
Calr 52-bp deletion
Flt3 ITD
Npm1c
Idh1 R132H
Dnmt3a R882H
Ezh2 KO
Utx KO
HSC
Immature prog.
Lymphoid prog.
Neutrophil prog.
Mono/DC prog.
MEP
MK prog.
Early Ery prog.
Mid Ery prog.
Late Ery prog.
Basophil prog.
Mast prog.
Unassigned
C
Procr (HSC)
Dntt (Lymphoid)
Pf4 (MK)
Klf1 (Erythroid)
Elane (Neutrophil)
Irf8 (Mono/DC)
Mcpt8 (Baso)
Gzmb (Mast)

Figure S1 (related to Figure 1). Reference-based data integration

(A) Expression of lineage marker genes in the reference atlas (44,802 cells). Color scales show the log-normalized expression. HSC, hematopoietic stem cell; MK, megakaryocyte; Mono, monocyte; DC, dendritic cell; Baso, basophil. (B) Mutant mouse hematopoietic landscapes projected onto the reference atlas. prog, progenitors; MEP, megakaryocyte-erythroid progenitors; Ery, erythroid. (C) Expression of lineage marker genes in the preleukemic mouse hematopoietic atlas (269,048 cells), showing successful data projection. Color scales show the log-normalized expression.

Figure S2

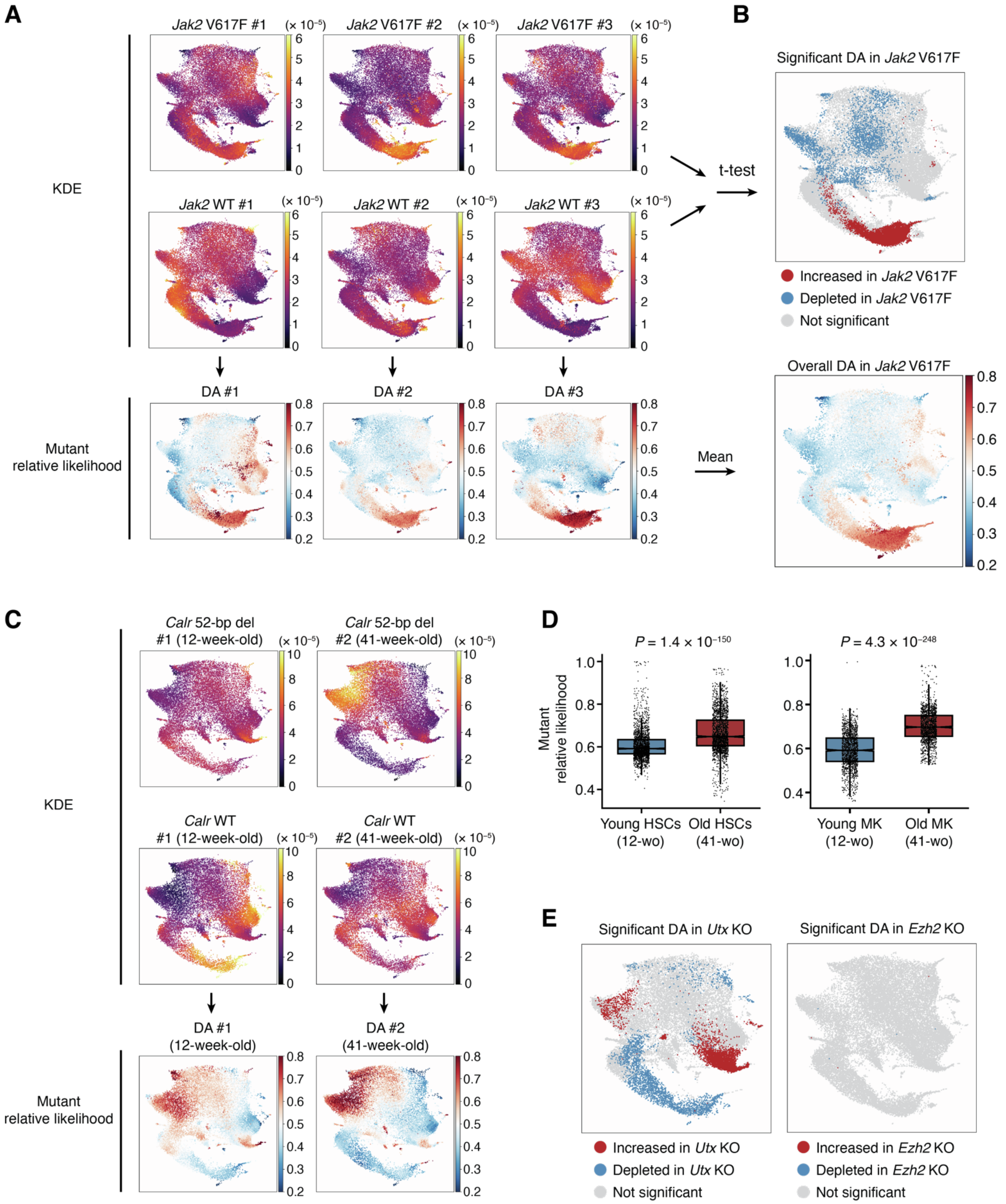
A
B
C
D
E
KDE
Mutant relative likelihood
Jak2 V617F #1
Jak2 V617F #2
Jak2 V617F #3
Jak2 WT #1
Jak2 WT #2
Jak2 WT #3
(× 10−5)
DA #1
DA #2
DA #3
t-test
Mean
Significant DA in Jak2 V617F
Increased in Jak2 V617F
Depleted in Jak2 V617F
Not significant
Overall DA in Jak2 V617F
Calr 52-bp del #1 (12-week-old)
Calr 52-bp del #2 (41-week-old)
Calr WT #1 (12-week-old)
Calr WT #2 (41-week-old)
DA #1 (12-week-old)
DA #2 (41-week-old)
P = 1.4 × 10−150
P = 4.3 × 10−248
Young HSCs (12-wo)
Old HSCs (41-wo)
Young MK (12-wo)
Old MK (41-wo)
Significant DA in Utx KO
Significant DA in Ezh2 KO
Increased in Utx KO
Depleted in Utx KO
Increased in Ezh2 KO
Depleted in Ezh2 KO

Figure S2 (related to Figure 2). Differential abundance in preleukemic mutant models

(A) Workflow of MELD-based differential abundance analysis. First, KDEs of the individual replicates of *Jak2* V617F model were computed, and differential abundance was quantified by performing cell-wise L1 normalization of the KDEs as implemented in the MELD package. The mutant relative likelihoods from all pairwise comparisons were averaged to obtain the mutation-specific differential abundance (DA) landscapes. (B) Statistically significant differential abundance in the *Jak2* V617F model. (C) KDEs and differential abundance landscapes for the two replicates (young and old) of *Calr* mutant model. (D) Significant difference in the mutant relative likelihood of young (12-week-old) and aged (41-week-old) HSCs (left) and megakaryocyte progenitors (right). Box plots show median and first/third quartiles. The whisker extends from the smallest to the largest values within 1.5 × IQR from the box hinges. *P*-values are from two-sided Wilcoxon signed-rank test. (E) Significant differential abundance in the *Utx* KO (left) and the *Ezh2* KO model (right).

Figure S3

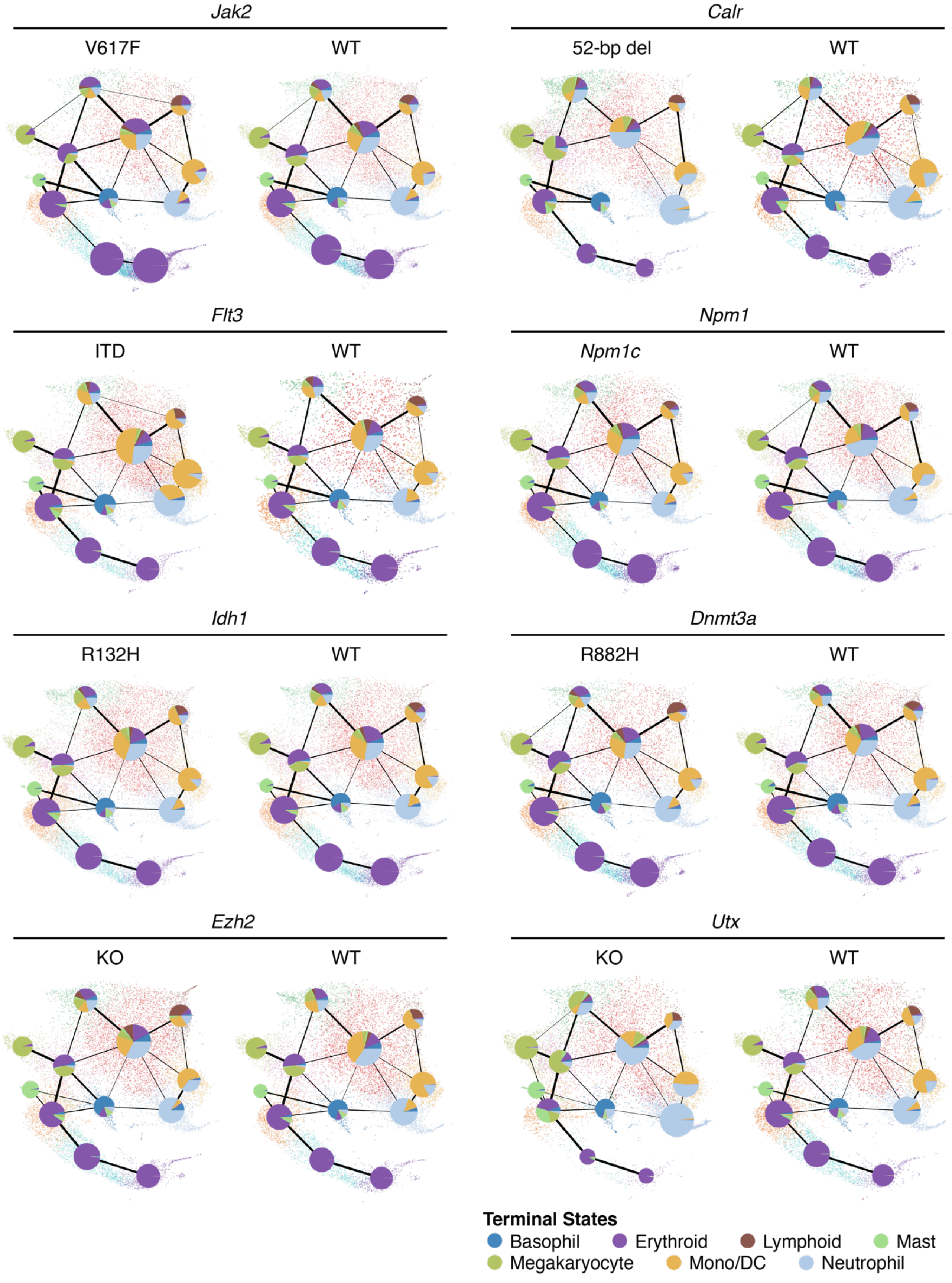

Jak2
V617F
WT
Calr
52-bp del
WT
Flt3
ITD
WT
Npm1
Npm1c
WT
Idh1
R132H
WT
Dnmt3a
R882H
WT
Ezh2
KO
WT
Utx
KO
WT
Terminal States
Basophil
Erythroid
Lymphoid
Mast
Megakaryocyte
Mono/DC
Neutrophil

Figure S3 (related to Figure 3). Biased cellular fate probability in preleukemic mutant models

CellRank-based fate probabilities from each cell type to the seven terminal states are visualized on the individual UMAP plots as pie charts with PAGA connections.

Figure S4

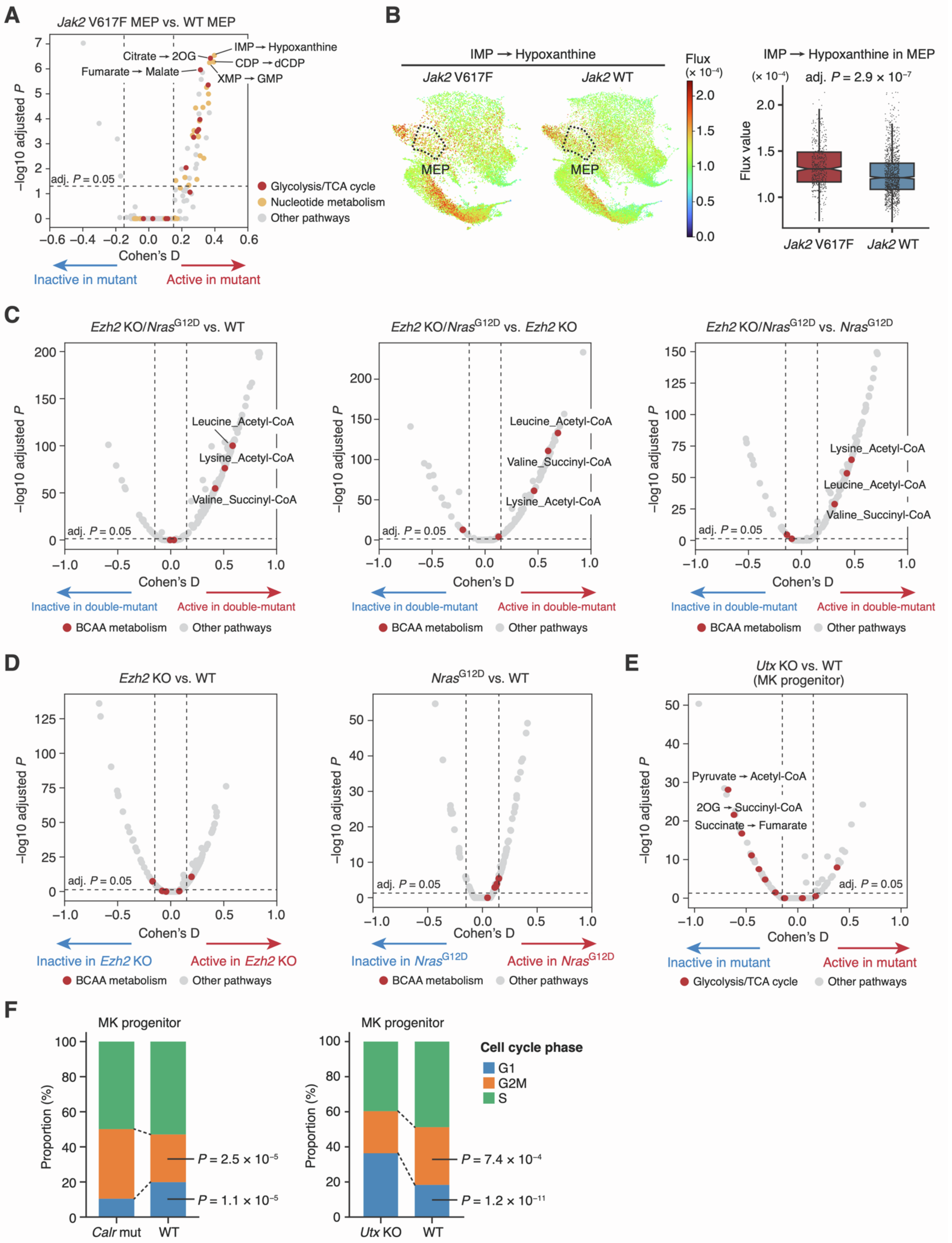
A
Jak2 V617F MEP vs. WT MEP
IMP → Hypoxanthine
Citrate → 2OG
CDP → dCDP
Fumarate → Malate
XMP → GMP
adj. P = 0.05
−log10 adjusted P
Cohen's D
Inactive in mutant
Active in mutant
Glycolysis/TCA cycle
Nucleotide metabolism
Other pathways
B
IMP → Hypoxanthine
Jak2 V617F
Jak2 WT
MEP
Flux (× 10−4)
IMP → Hypoxanthine in MEP
adj. P = 2.9 × 10−7
Flux value
C
Ezh2 KO/NrasG12D vs. WT
Ezh2 KO/NrasG12D vs. Ezh2 KO
Ezh2 KO/NrasG12D vs. NrasG12D
Leucine_Acetyl-CoA
Lysine_Acetyl-CoA
Valine_Succinyl-CoA
Inactive in double-mutant
Active in double-mutant
BCAA metabolism
Other pathways
D
Ezh2 KO vs. WT
NrasG12D vs. WT
Inactive in Ezh2 KO
Active in Ezh2 KO
Inactive in NrasG12D
Active in NrasG12D
E
Utx KO vs. WT (MK progenitor)
Pyruvate → Acetyl-CoA
2OG → Succinyl-CoA
Succinate → Fumarate
F
MK progenitor
Proportion (%)
Cell cycle phase
G1
G2M
S
P = 2.5 × 10−5
P = 1.1 × 10−5
Calr mut
WT
P = 7.4 × 10−4
P = 1.2 × 10−11
Utx KO

Figure S4 (related to Figure 4). Literature-based validation of transcriptome-based metabolic profiling

(A) A volcano plot comparing the metabolic activities of *Jak2* mutant and wild-type MEPs. The x and y axes represent Cohen's D standardized difference of means and −log10 adjusted *P*-values, respectively. Each dot represents each metabolic reaction module and is colored according to the functional pathway. The five most significant modules from the glycolysis/TCA cycle or nucleotide synthesis pathways are indicated. The horizontal dotted line indicates the adjusted *P*-value of 0.05; the vertical dotted lines indicate the Cohen's D values of −0.15 and 0.15. (B) The cellular flux estimates of the most significantly altered metabolism of inosine monophosphate into hypoxanthine. The left panel shows the cellular level activities of this metabolic reaction. The right panel shows the significant upregulation of this reaction in *Jak2* mutant MEPs. Box plots show median and first/third quartiles. The whisker extends from the smallest to the largest values within 1.5 × IQR from the box hinges. *P*-values are from logistic regression and likelihood ratio test and are BH-adjusted. (C and D) Differential metabolic activities of the BCAA pathway in *Ezh2* KO/*Nras* G12D (double mutant), *Ezh2* KO, *Nras* G12D and WT mice from a published scRNA-seq dataset[32]. The double-mutant was compared to each of the other genotypes (C) and the single mutant models were compared to the wild-type (D). The whole c-Kit$^+$ HSPC populations from 4-month-old mice were compared. The metabolic reactions in the BCAA pathway are colored red. (E) A volcano plot comparing glycolysis and TCA cycle activities in the *Utx* KO and WT megakaryocyte progenitors. (F) Cell cycle phase of *Calr*-mutant (left) and *Utx* KO (right) megakaryocyte progenitors. *P*-values are from Fisher's exact test.

Figure S5

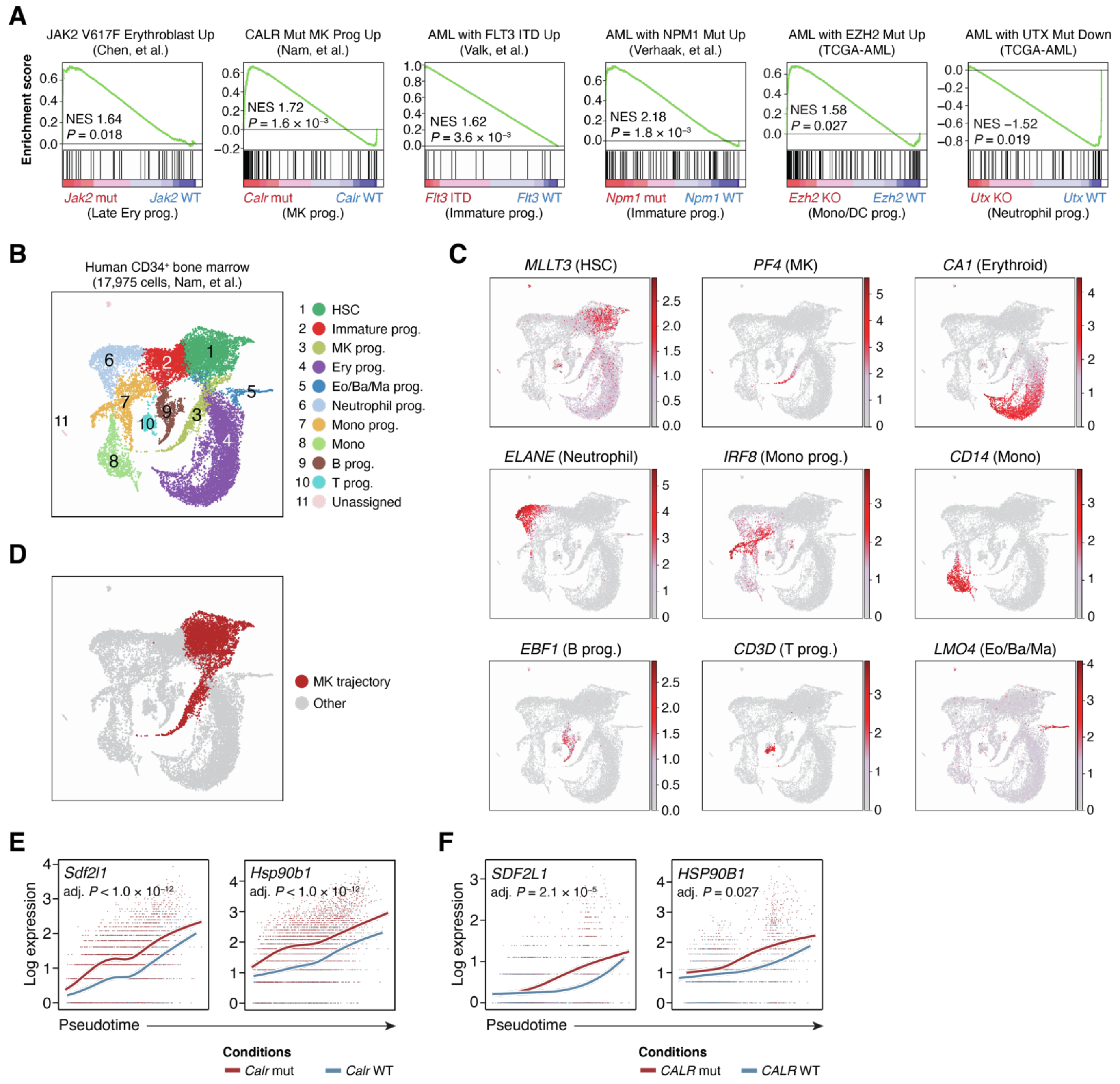

Figure S5 (related to Figure 5). Consistent gene expression changes in mouse models and human patients

(A) Significant enrichment of patient-derived gene signatures in our mouse models. Patient-derived gene signatures were obtained from previous reports[36–39] for *JAK2*, *CALR*, *FLT3* and *NPM1* mutations and from differential expression analysis of TCGA AML data[1] for *EZH2* and *UTX* mutations. For *EZH2* and *UTX* signatures, patients with mutations and copy number loss in each gene were compared to all other patients using DESeq2 and significantly upregulated genes (log2 fold change >1.0 and BH-adjusted P <0.1) and downregulated genes (log2 fold

change <−1.0 and BH-adjusted *P* <0.1) were used for enrichment analysis. The upregulated gene set in *UTX* mutated patients was not evaluable with GSEA due to the small gene set size (<15 genes). NES, normalized enrichment score. (B) UMAP plot of $CD34^+$ bone marrow HSPCs from patients with *CALR*-mutated essential thrombocythemia (17,975 cells)[37]. HSC, hematopoietic stem cell; prog, progenitors; MK, megakaryocyte; Ery, erythroid; Eo/Ba/Ma, eosinophil/basophil/mast cell; Mono, monocyte. (C) Expression of lineage marker genes. Color scales show the log-normalized expression. (D) Megakaryocyte differentiation trajectory in patients with *CALR*-mutated essential thrombocythemia. (E and F) Consistent pseudotemporal gene expression patterns of unfolded protein response genes in our mouse model (E) and patients (F). *P*-values are from Wald test and are BH-adjusted.

Figure S6

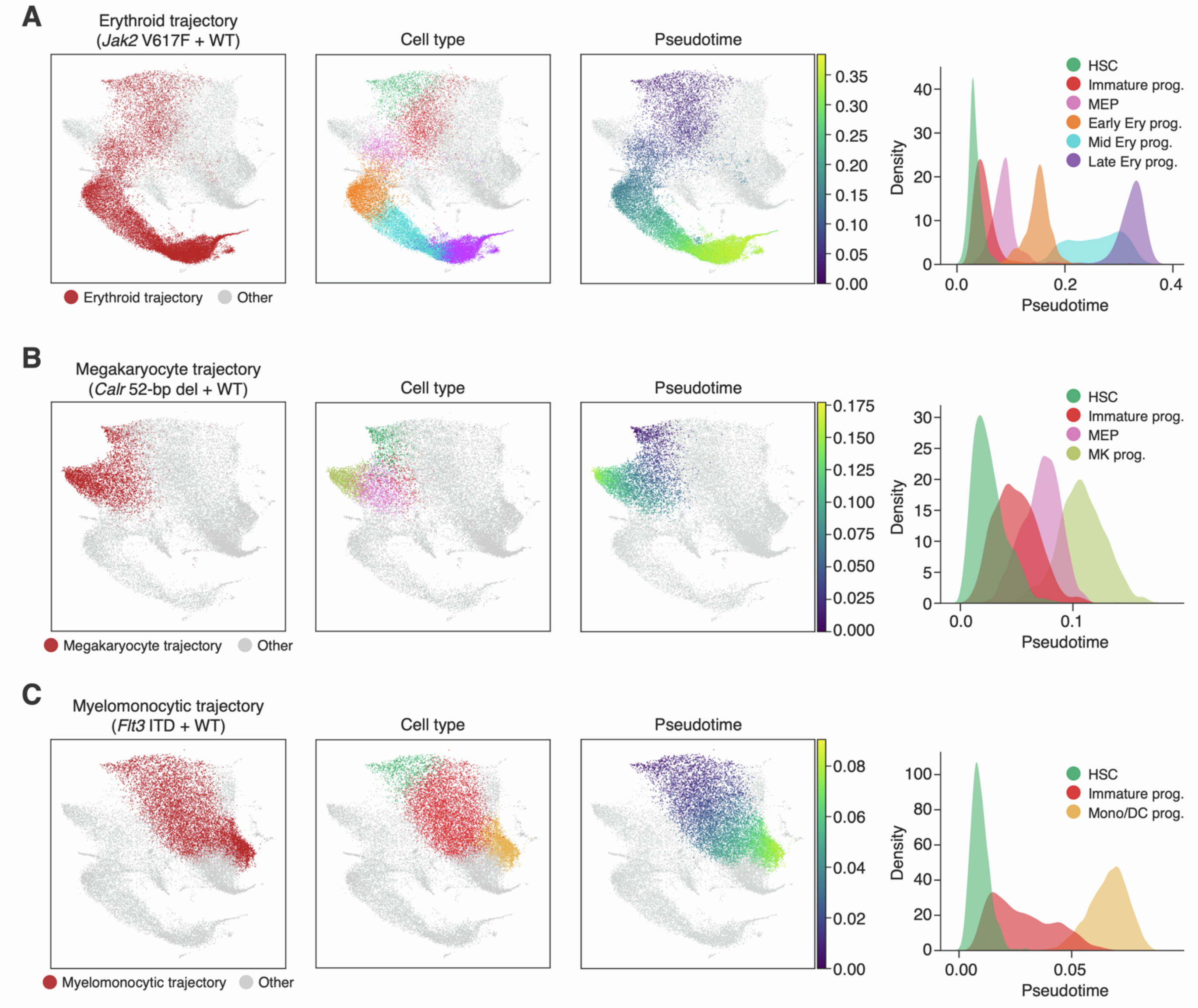

Figure S6 (related to Figure 5). Defining the lineage trajectories for pseudotemporal gene expression analysis

(A-C) The *Jak2* mutant and wild-type erythroid trajectory (A), the *Calr* mutant and wild-type megakaryocyte trajectory (B), and the *Flt3* mutant and wild-type myelomonocytic trajectory (C) were defined based on the lineage fate probability (see Methods). The defined lineage trajectories, cell types and pseudotime are shown on the UMAP plots. The right panels show the pseudotime distribution of each cell type, defining the pseudotime ranges of dominant cell types in each trajectory.

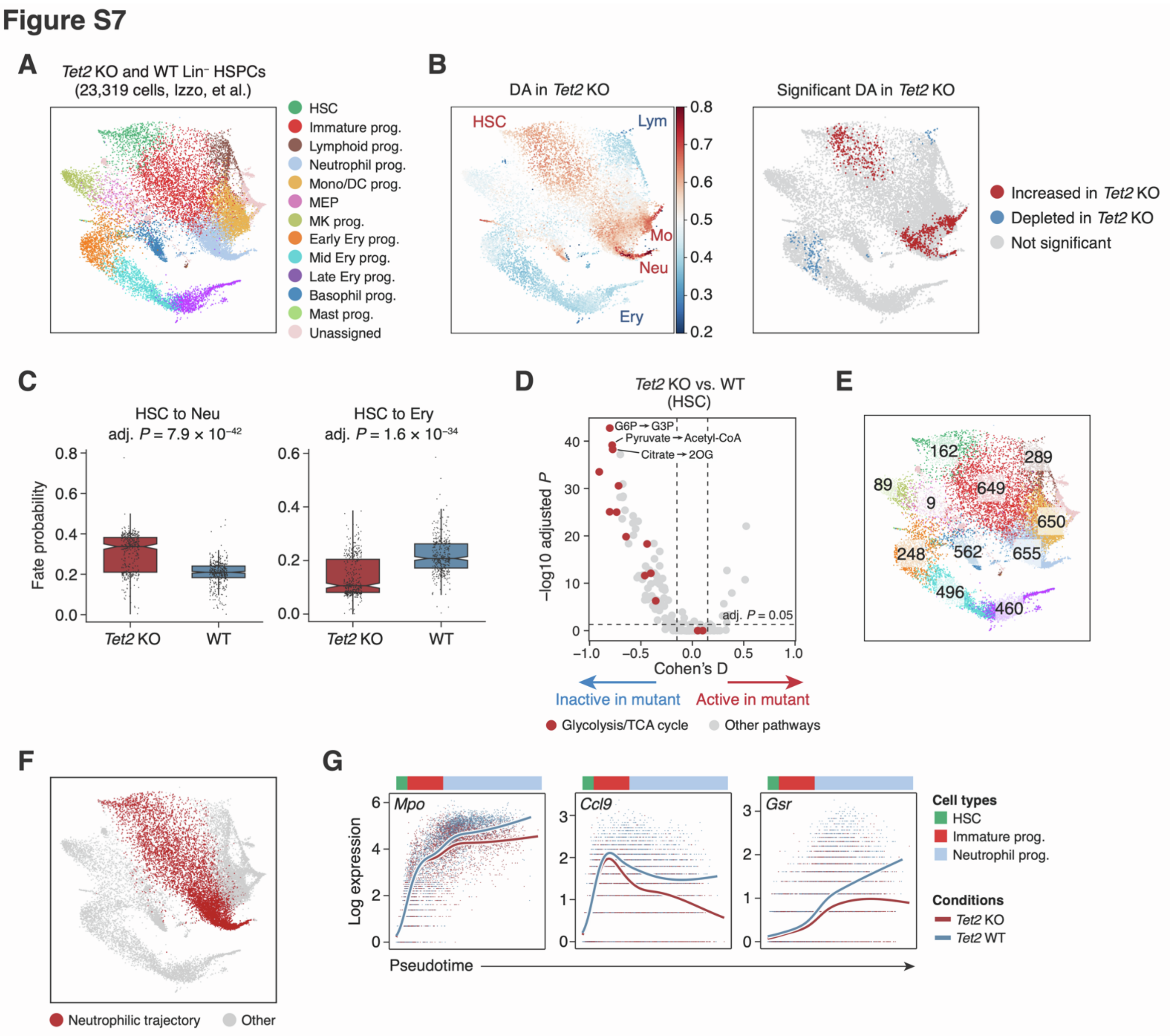

Figure S7 (related to Figure 1-5). Preleukemic perturbations in *Tet2* KO mouse HSPCs

(A) A previously published scRNA-seq dataset of *Tet2* KO mouse HSPCs[42] projected onto our reference atlas. prog, progenitors; MEP, megakaryocyte-erythroid progenitors; Ery, erythroid. (B) Differential abundance (DA) landscape (left) and statistically significant DA (right) in the *Tet2* KO model. Cell types with the highest median mutant relative likelihoods are indicated. (C) Significant differences in the neutrophilic (left) and erythroid (right) probability between *Tet2* KO and wild-type HSCs. Box plots show median and first/third quartiles. The whisker extends from the smallest to the largest values within 1.5 × IQR from the box hinges. *P*-values are from logistic regression and likelihood ratio test and are BH-adjusted. (D) A volcano plot comparing the metabolic activities of *Tet2* KO and wild-type HSCs. Each dot represents each metabolic reaction module and is colored according to the functional pathway. The horizontal

dotted line indicates the adjusted *P*-value of 0.05; the vertical dotted lines indicate the Cohen's D values of −0.15 and 0.15. (E) The number of genes differentially expressed in each cell type of the *Tet2* KO and wild-type mice. (F) The *Tet2* KO and wild-type neutrophilic trajectory. The neutrophilic trajectory was defined as the cells belonging to the 'HSC', 'Immature prog' or 'Neutrophil prog' clusters and with the neutrophilic fate probability ≥0.15 and the pseudotime <0.30. (G) Significantly altered gene expression patterns in the *Tet2* KO and wild-type neutrophilic trajectory. The pseudotime ranges of dominant cell types are indicated with colored bars.

Figure S8

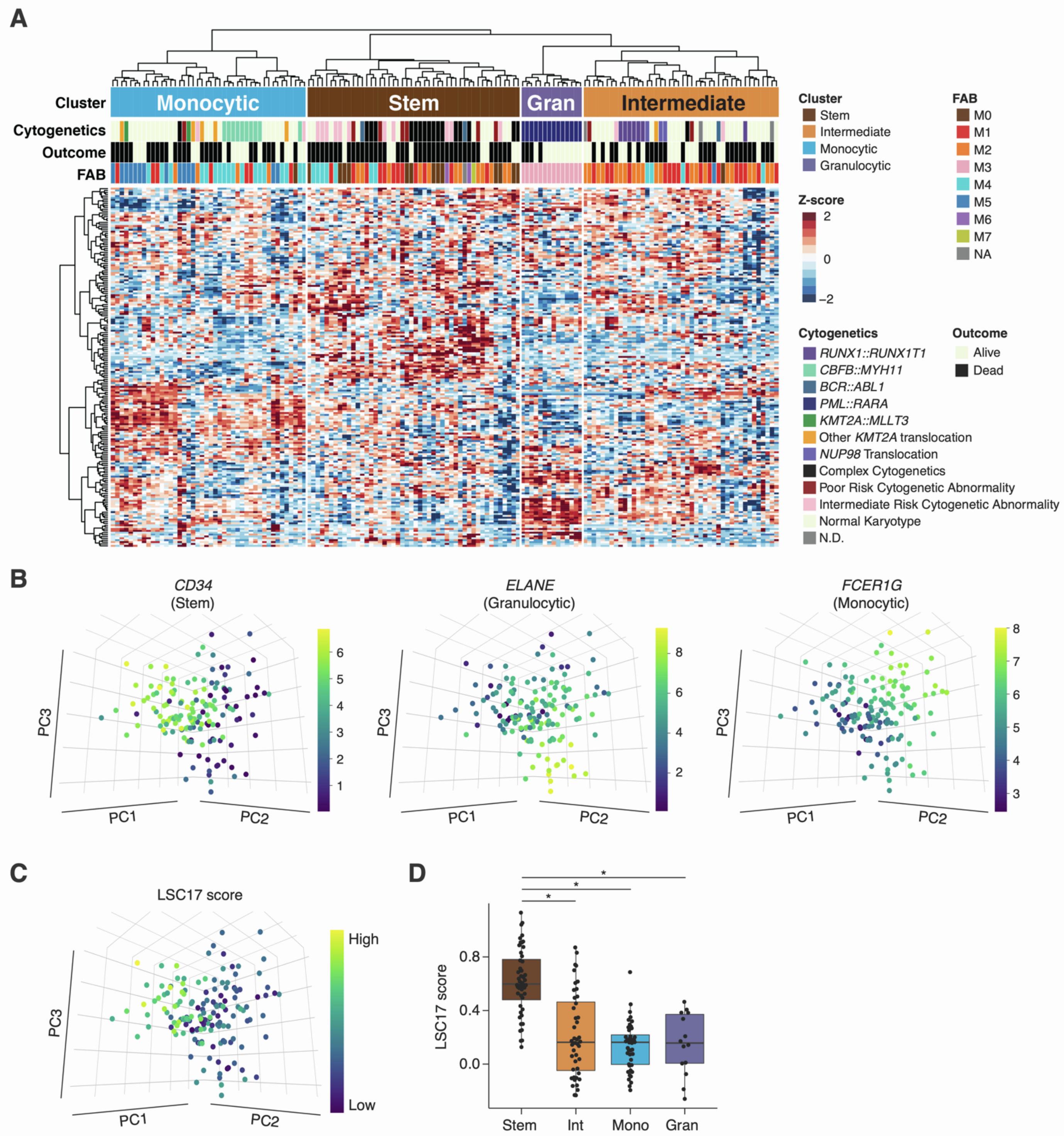

Figure S8 (related to Figure 6). Clustering of TCGA AML patients

(A) Hierarchical clustering of the TCGA AML cohort[1] using the 216 PLPS genes. (B) Characteristic expression of differentiation marker genes in the TCGA AML samples. Color scales show the log2 TPM expression values. (C) LSC17 scores[51] in the TCGA AML cohort. The Color scale shows the LSC17 scores. (D) LSC17 scores in different clusters of TCGA

AML patients. Int, Intermediate; Mono, Monocytic; Gran, Granulocytic. Box plots show median and first/third quartiles. The whisker extends from the smallest to the largest values within 1.5 × IQR from the box hinges. *P*-values are from the two-sided Wilcoxon rank-sum test. **P* <1.0 × 10^{-7}.

Figure S9

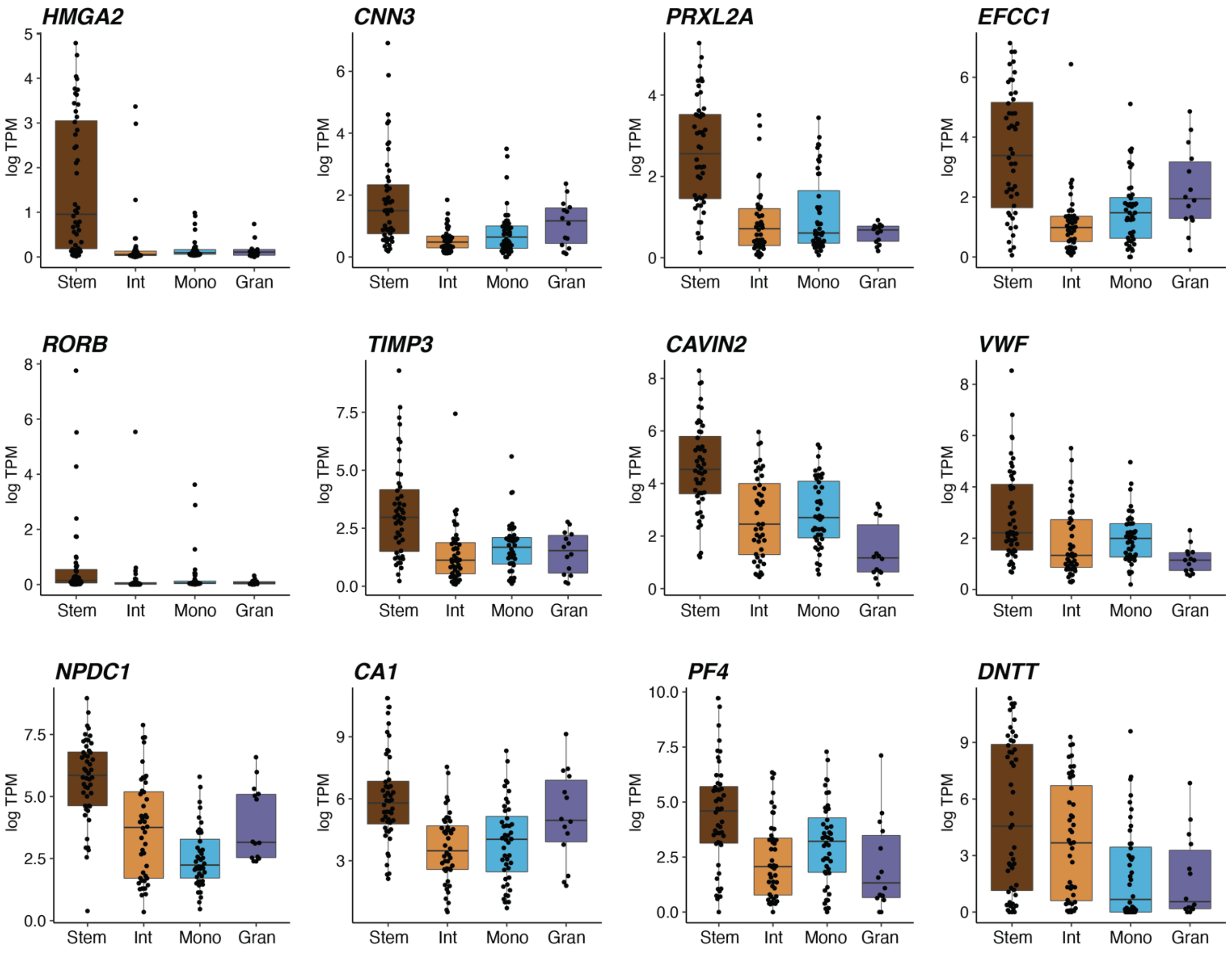

Figure S9 (related to Figure 6). Significant upregulation of 12 PLPS genes in the Stem cluster

Expression levels (log2 TPM) of 12 genes significantly upregulated in the Stem cluster (BH-adjusted *P* <0.05 and log2 fold change >2). Int, Intermediate; Mono, Monocytic; Gran, Granulocytic. Box plots show median and first/third quartiles. The whisker extends from the smallest to the largest values within 1.5 × IQR from the box hinges.

Figure S10

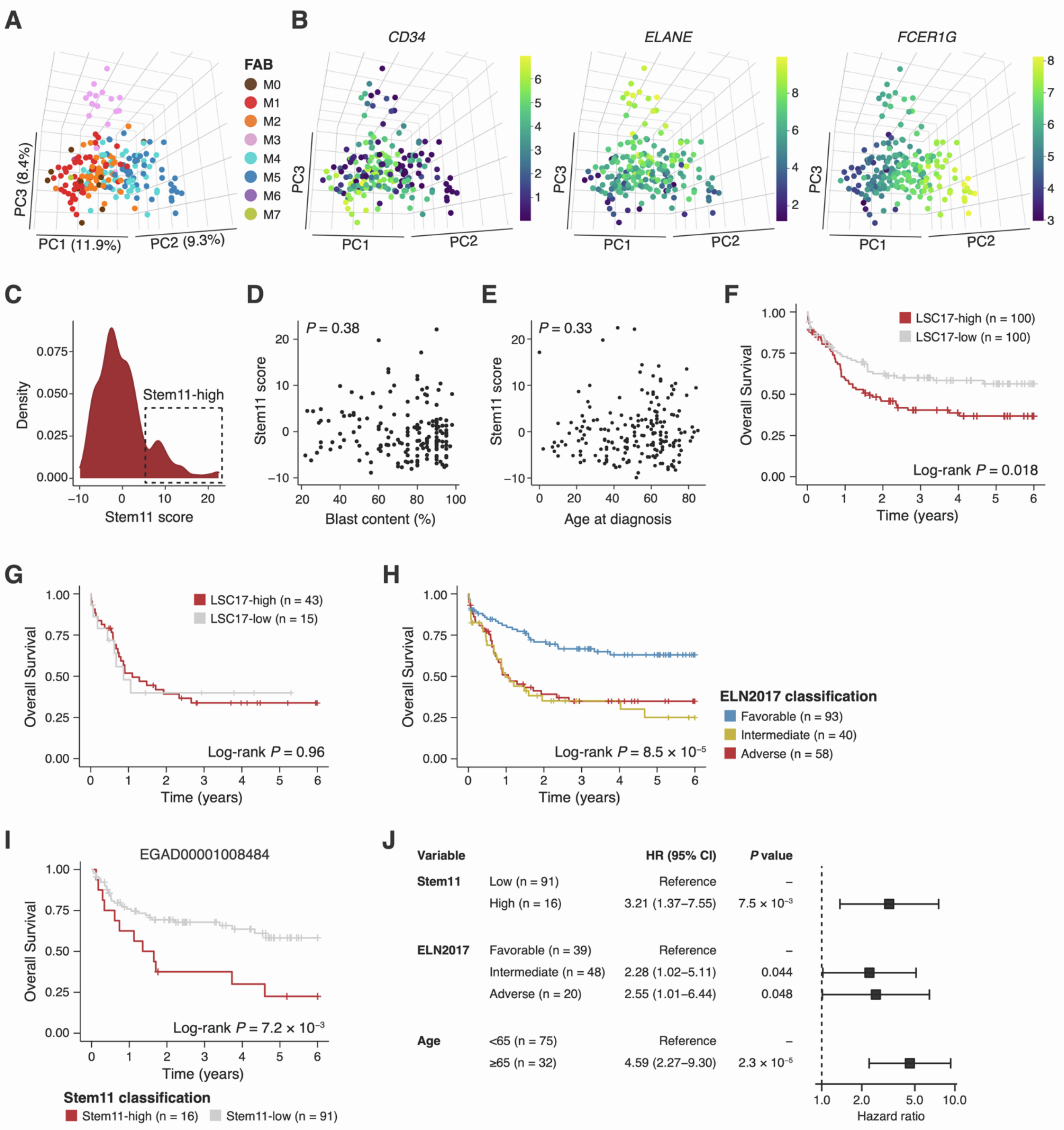

Figure S10 (related to Figure 6). PLPS and Stem11-based characterization of AML patient cohorts

(A and B) Principal component analysis of the Beat AML cohort. Samples are colored according to the FAB classification (A) and the expression levels (log2 TPM) of differentiation marker genes (B). PC1, PC2 and PC3 represent the first three principal components with the

percentage of variance explained indicated on the axes. (C) A density plot showing the distribution of Stem11 scores in the Beat AML cohort. The threshold for Stem11-high patients is indicated by a dashed rectangle. (D and E) Scatter plots comparing Stem11 scores to blast content (D) and patient age (E). Pearson correlation *P*-values are indicated. (F and G) Survival analysis comparing the LSC17 high and low patients among the entire Beat AML cohort (F) and the ELN2017 adverse risk patients in the Beat AML cohort (G). (H) Survival analysis comparing the ELN2017 risk groups in the Beat AML cohort. (I) Survival analysis comparing the Stem11 high and low groups in the EGAD00001008484 AML dataset[55]. (J) Multivariate Cox proportional hazards analysis for overall survival in the EGAD00001008484 cohort. Stem11 classification, ELN2017 classification and patient age were included in the model. Error bars show the 95% confidence interval. HR, hazard ratio; CI, confidence interval.

Figure S11

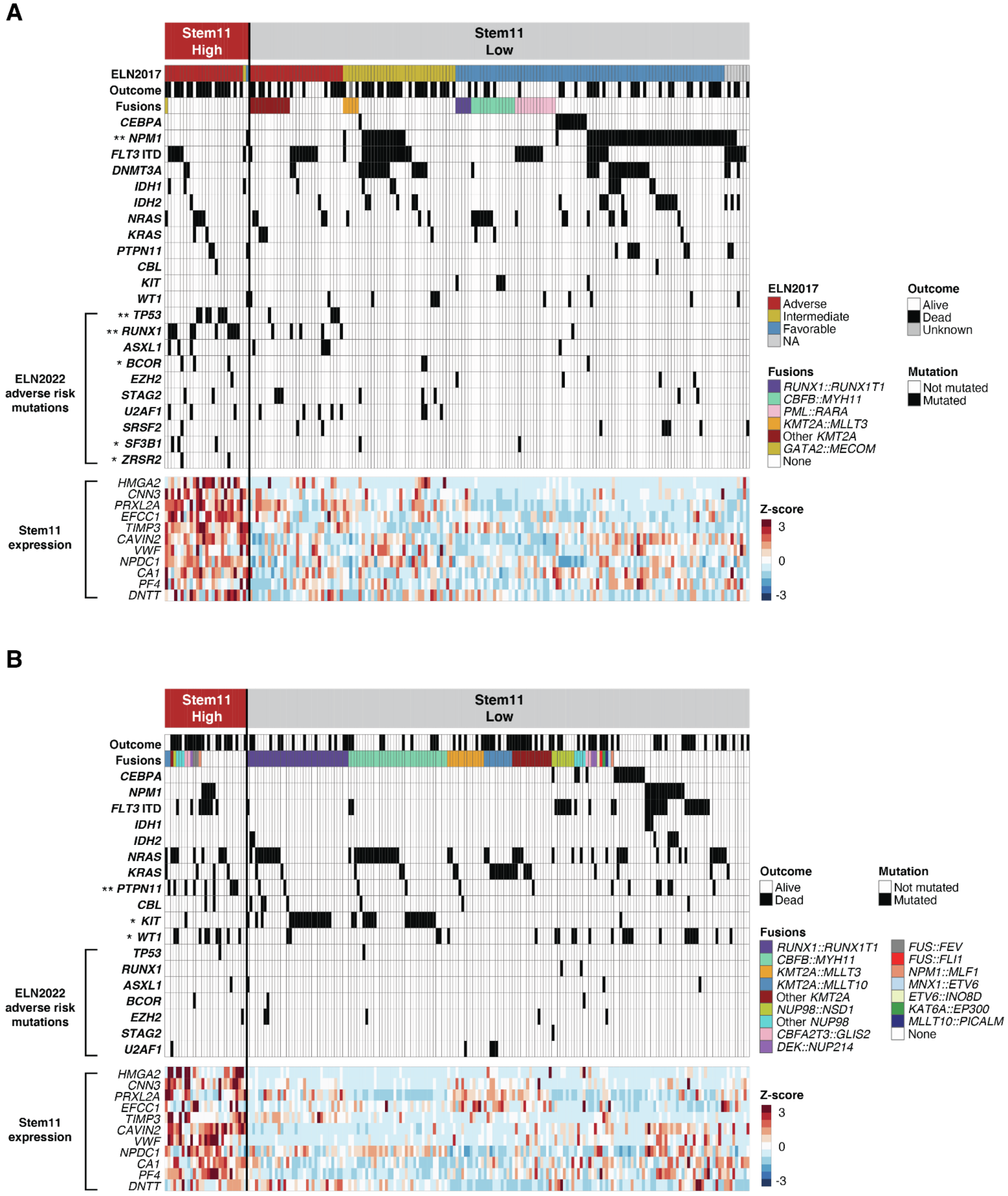

Figure S11 (related to Figure 6). Mutational landscapes of Stem11 high and low patients

(A and B) The mutational landscapes of the Beat AML cohort (A) and the TARGET pediatric AML cohort (B). Each column represents a single patient. The upper panels show the

presence of individual mutations in each patient. The lower panels show the expression heatmaps of Stem11 genes. Genes with significantly different prevalence between Stem11 high and low patients are denoted with asterisks (*). *P*-values were derived from Fisher's exact test. *P <0.05; **P <0.001.

Figure S12

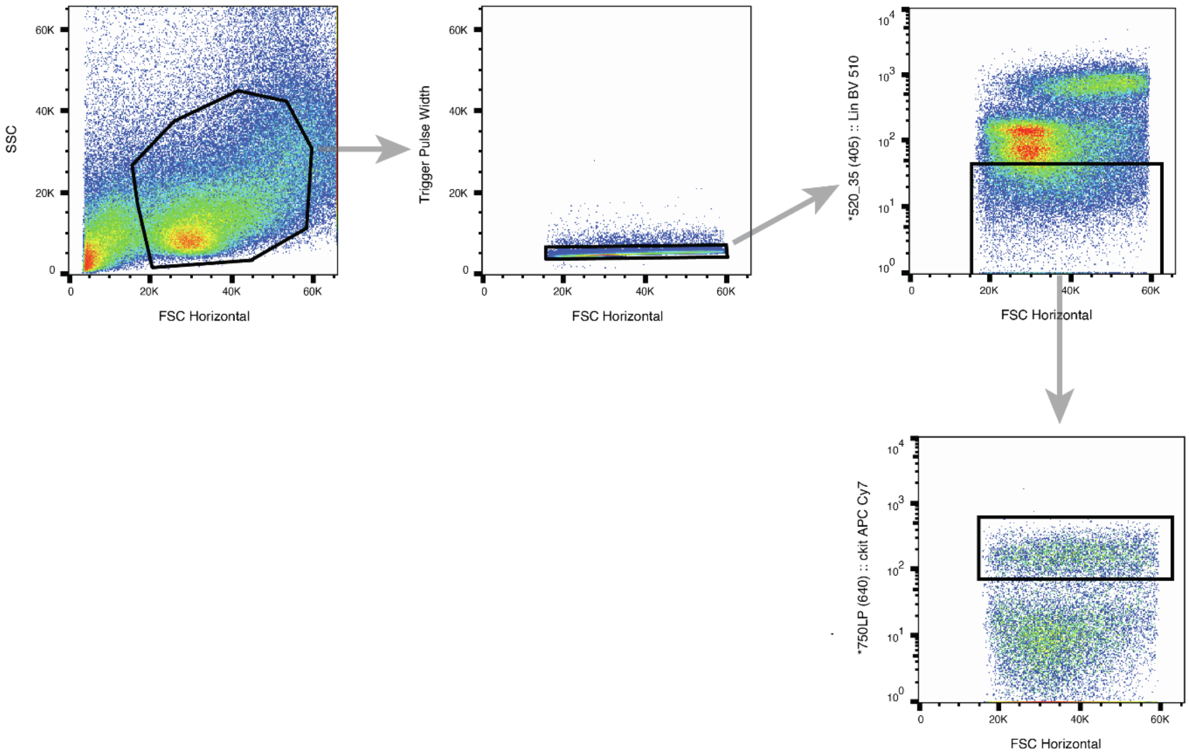

Figure S12 (related to Figure 1-6). Flow cytometry sorting of mouse HSPCs

Representative gating strategy to sort mouse bone marrow Lineage^{-} c-Kit^{+} HSPCs.
